# Immunomodulatory effects of renin–angiotensin system inhibitors on T lymphocytes in mice with colorectal liver metastases

**DOI:** 10.1136/jitc-2019-000487

**Published:** 2020-05-24

**Authors:** Dora Lucia Vallejo Ardila, Katrina A Walsh, Theodora Fifis, Rita Paolini, Georgios Kastrappis, Christopher Christophi, Marcos Vinicius Perini

**Affiliations:** Surgery, The University of Melbourne Faculty of Medicine Dentistry and Health Sciences, Melbourne, Victoria, Australia

**Keywords:** translational medical research, lymphocytes, tumor-infiltrating, immunotherapy, immunomodulation, immunohistochemistry

## Abstract

**Background:**

It is now recognized that many anticancer treatments positively modulate the antitumor immune response. Clinical and experimental studies have shown that inhibitors of the classical renin–angiotensin system (RAS) reduce tumor progression and are associated with better outcomes in patients with colorectal cancer. RAS components are expressed by most immune cells and adult hematopoietic cells, thus are potential targets for modulating tumor-infiltrating immune cells and can provide a mechanism of tumor control by the renin–angiotensin system inhibitors (RASi).

**Aim:**

To investigate the effects of the RASi captopril on tumor T lymphocyte distribution in a mouse model of colorectal liver metastases.

**Methods:**

Liver metastases were established in a mouse model using an autologous colorectal cancer cell line. RASi (captopril 750 mg/kg) or carrier (saline) was administered to the mice daily via intraperitoneal injection, from day 1 post-tumor induction to endpoint (day 15 or 21 post-tumor induction). At the endpoint, tumor growth was determined, and lymphocyte infiltration and composition in the tumor and liver tissues were analyzed by flow cytometry and immunohistochemistry (IHC).

**Results:**

Captopril significantly decreased tumor viability and impaired metastatic growth. Analysis of infiltrating T cells into liver parenchyma and tumor tissues by IHC and flow cytometry showed that captopril significantly increased the infiltration of CD3^+^ T cells into both tissues at day 15 following tumor induction. Phenotypical analysis of CD45^+^ CD3^+^ T cells indicated that the major contributing phenotype to this influx is a CD4 and CD8 double-negative T cell (DNT) subtype, while CD4^+^ T cells decreased and CD8^+^ T cells remained unchanged. Captopril treatment also increased the expression of checkpoint receptor PD-1 on CD8^+^and DNT subsets.

**Conclusion:**

Captopril treatment modulates the immune response by increasing the infiltration and altering the phenotypical composition of T lymphocytes and may be a contributing mechanism for tumor control.

## Background

Colorectal cancer (CRC) is the third most common cancer diagnosis, and it is ranked second as cancer causing death for both sexes combined in high-income countries.^1^ Over half of the patients with CRC will develop metastatic liver disease. Subsequently, colorectal liver metastases (CRLM) result in death in at least two-thirds of these patients.^2^ Surgical resection of CRLM is the only potential curative treatment and, when combined with chemotherapy, can achieve 5-year survival rates of 40%–60%.^2 3^ However, the majority of these patients will develop tumor recurrence, most commonly in the liver.^4^ Current research to improve this clinical outcome is focusing on identifying reliable prognostic indicators of treatment response,^5 6^ tumors with a high-risk molecular signature for recurrence,^7^ and in developing novel strategies to immunomodulate the tumor microenvironment (TME).^8^ Established tumors subvert the host antitumor immune response through several mechanisms, including the accumulation of immunosuppressive tumor-associated macrophages (TAMs), myeloid-derived suppressive cells (MDSCs) and cancer-associated fibroblasts (CAFs). Also, the production of immunosuppressive factors influences the infiltration and activation of effector immune cells and the upregulation of T cell inhibitory ligands like programmed death-ligand 1 (PD-L1) on tumor, stromal and immune cells.^9^ During the last few years, many different cancer types have proven responsive to therapeutic targeting of inhibitory T cell receptors by immune check-point inhibitors, such as ipilimumab,^10^ nivolumab^11^ and pembrolizumab.^12^ In the case of CRLM, only a small subset of patients, those with deficient mismatch repair pathways, respond to these treatments.^13^ Targeting immunological features that promote metastatic growth, such as molecules that are proinflammatory, proangiogenic, profibrotic, or specific leukocyte subsets that may change in number, phenotype or function in different types of cancer,^14–16^ could tip the balance towards an antitumor immune response.

Renin- angiotensin inhibitors (RASi) have shown antitumor effects that may have a profound impact in cancer therapy.^17–19^ The role of the various RASi in modulating inflammation^20^ and fibrosis^21 22^ is well documented. Renin–angiotensin system (RAS) pathway components are expressed by most cells of the innate and adaptive immune system.^23^ *In vitro* studies indicate that RAS signaling modulates the activity of various immune cells; on the other hand,*in vivo* studies of cancer immune responses modulated by RASi are still scarce. One study using a mouse renal cancer model reported that captopril increased tumor growth and inhibited the antigen-specific activation of CD8^+^ and CD4^+^ T cells while promoting antigen-specific B cells and their infiltration into tumors.^24^ In contrast, studies from this laboratory using captopril or angiotensin receptor 1 (AT1R) blockers reported a significant reduction in CRLM and modulation of TAMs.^18 19 25 26^ Supporting these findings, Nakamura *et al* demonstrated that RASi reduced the immunosuppressive activity of TAMs, MDSCs, and CAFs in the TME, leading to the induction and tumor infiltration of tumor antigen-specific T cells.^27^

In this study, the effects of captopril treatment (RASi) on T lymphocyte subtypes and their expression of some activation or inhibitory factors were investigated in a CRC liver metastasis mouse model.

## Materials and methods

### Animals and experimental model of CRLM

**Figure 1:**
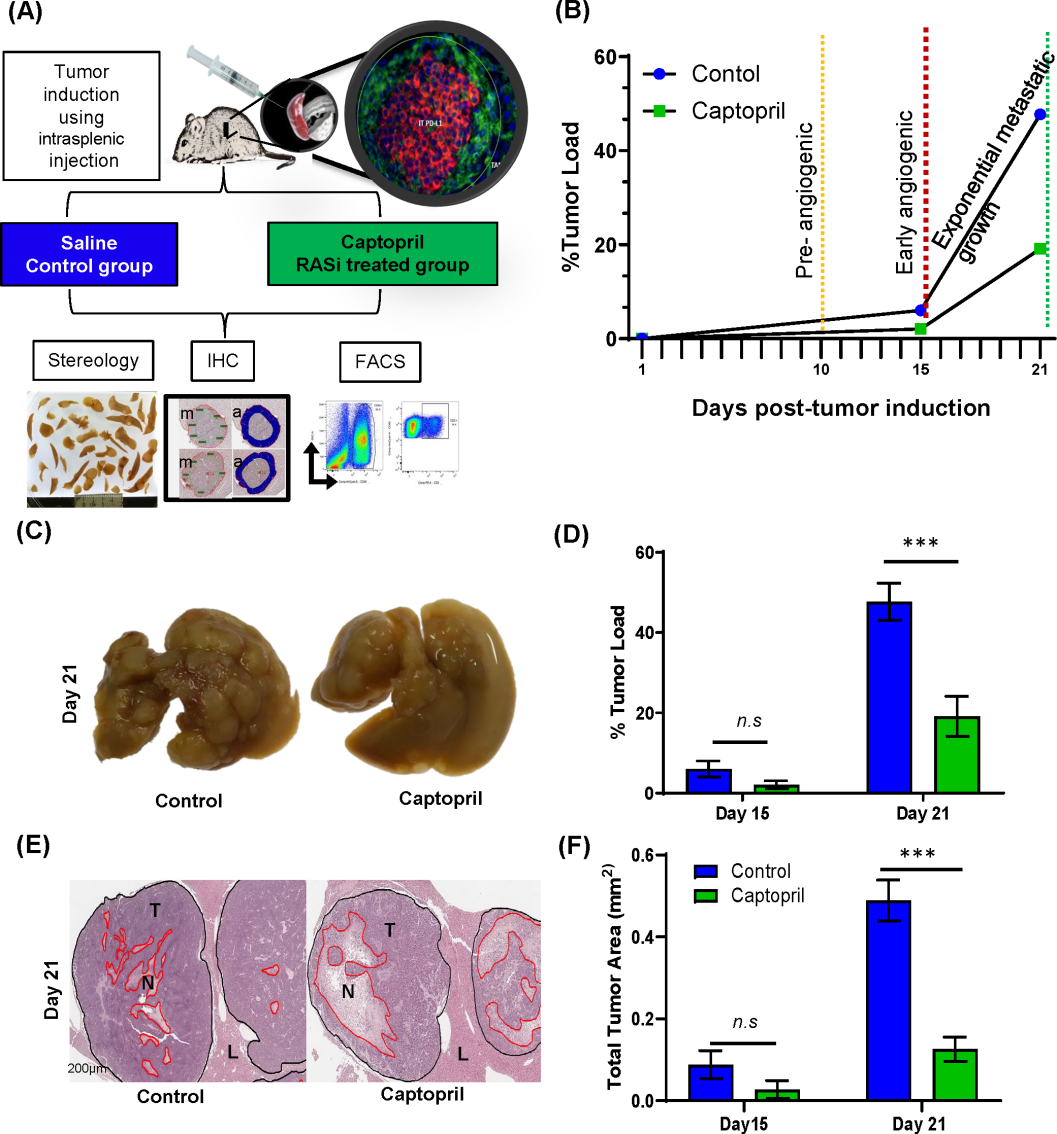
Control of tumor growth and viability by RASi treatment. (A) Liver metastases were induced by intrasplenic tumor induction and allowed to develop for 15 and 21 days. Captopril or saline control treatment was delivered daily by via intraperitoneal injection. At each endpoint, mice were terminally anesthetized, and their livers perfused with saline prior to removal. Most of the livers were fixed and prepared for IHC and stereology, while a small proportion was prepared for flow cytometry, including the separation of tumor from the liver tissues. (B) Schematic of tumor growth kinetics following tumor induction and daily treatment. (C) Representative livers of each treatment are pictured. (D) Livers were fixed, sectioned and analyzed by stereology for % tumor burden of the total liver area and (E) stained with H&E for viable tumor analyses (T, tumor; N, necrosis and L, Liver). (F) Proportion of tumor viability. Data represent average ±SEM of n=5 per group; significance was calculated by Student t-test. ***P<0.001. FACS, fluorescent-activated cell sorting; IHC, immunohistochemistry; RASi, renin–angiotensin system inhibitor; n.s, not significant.

Male CBA mice (ARC, Perth) were maintained in standard cages with irradiated food and water supplied *ad libitum*. The primary cancer cell line mouse CRC cell (MoCR) was derived from a dimethyl hydrazine-induced primary colon carcinoma in the CBA mouse and maintained *in vivo* by serial passage in the flanks of CBA mice.^28^ For passage and experimentation, subcutaneous tumors were teared apart, passed through a filter, treated with Ethylenediaminetetra-acetic acid (EDTA) and washed in phosphate-buffered saline (PBS) to make a single cell suspension. CRLMs were induced by intrasplenic injection of 5×10^4^ tumor cells followed by splenectomy. Metastases are fully established by 21 days following tumor induction (figure 1A, B).

### Experimental design

Tissues were collected at days 15 and 21 post-tumor induction and were used to evaluate tumor growth and Tcell immune infiltration by immunohistochemistry (IHC) and fluorescent-activated cell sorting (FACS) (figure 1A).

### Treatment protocol

Mice received tumor induction and were separated into two groups: control (saline) and treatment (captopril). Captopril (pH adjusted to 7.4) was administered daily via intraperitoneal injection adjusted to the body weight of each mouse (750 mg/kg), from the day 1 post-tumor induction to endpoint. Control mice received an equal volume of saline. Tumor growth was assessed by stereology on day 15 or 21 following tumor induction.

At each endpoint, animals were terminally anesthetized; laparotomy was performed; and the abdominal cavity was examined for indications of extrahepatic metastases, paying close attention to the splenic bed. The liver was carefully perfused with 20 mL saline solution, then excised and harvested. Immediately following excision, liver weights were recorded. Livers were then fixed in formalin (10%) (Sigma Aldrich, Castle Hill, New South Wales, Australia) for 24 hours and then transferred to 70% ethanol.

### Stereology

Images of the whole liver were taken to examine the tumor distribution and burden load. The liver was then transversely sliced into sections of 1.5 mm thickness using a tissue fractionator. For large livers (saline control), every second slice was sampled for analysis. For smaller livers (captopril-treated tumors), every slice was taken for analysis. Liver slices were placed on a clear plastic plate; a digital camera (Nikon Coolpix 5000, E500) was used to capture the images, and these were analyzed using image analysis software (Image-Pro Plus, Perth, Australia).

Tumors were visualized as distinctive white/cream-colored areas against the red/brown liver tissue. Each tumor outline was traced using image analysis software to determine the area occupied by the tumor. Stereology technique was used to determine tumor burden.

### Immunohistochemistry

Formalin-fixed paraffin tissue sections (4 µm) were used with an indirect peroxidase labeling technique (Envision Plus, DAKO, Australia). Following deparaffinization and rehydration, endogenous peroxidase activity was blocked with 3% H_2_O_2_, and non-specific binding inhibited with 10% normal goat serum (01–6201 Thermo Fisher scientific). Heat-induced epitope retrieval was used. Antigens were visualized by immunohistochemical staining using their respective antibodies diluted as follows: CD3 1:100 (Clone A0452 DAKO), CD4 1:1000 (4SM95 e-Bioscience), PD-L1 1:500 (E1L3N, Cell Signaling Technology) and negative controls were stained without the corresponding primary antibodies. Following primary antibody treatment, sections were incubated with a horseradish peroxidase-labeled polymer secondary antibody. The antigen–antibody complex was visualized by diaminobenzene peroxidase substrate solution (DAKO, Australia). Viable tumor was determined by Haemotoxylin and Eosin (H&E) staining.

### Image caption and analysis algorithm

Slides were scanned at ×20 magnification (Aperio Scanscope AT Turbo, Leica Biosystems). Image analysis software (Aperio ImageScope) measured the number of positive cells within designated areas. Given the size of immune cells, the Positive Pixel Count v9 (V.9.1) algorithm was applied as a basis for detecting immune marker positivity, with the intensity thresholds adjusted manually to remove background artifacts and to account for variable differences in cell size (hue value (center): 0.1, hue width: 0.33 and color saturation threshold: 0.15). CD3^+^ cell distribution between tumor periphery and core was examined at day 15 (online supplementary figure S1A) and day 21 post-tumor induction. Same conditions were applied to measure CD4^+^ cells at day 15 (online supplementary figure S1B). The tumor ‘periphery’ was defined to include approximately an equal length of the leading edge of tumor cells and the adjacent stromal interface defined as the inner invasive margin. The ‘core of the tumor’ was defined as the total tumor area excluding the periphery. For each of these regions (periphery/leading edge or core), a total area up to of 100 µm per pixel containing the highest density of IHC^+^ cells was drawn to encompass these areas. When the inner invasive tumor margin (TM) or center of the tumor was smaller than 5 µm per pixel, the positivity of the whole tumor and the invasive margin was considered the same. PD-L1 expression was counted in the membranous and cytoplasmic tumor region using the same staining intensity thresholds (intensity threshold weak: 220–175, medium: 175–100, strong: 100–0, and negative pixels: −1).^29^

10.1136/jitc-2019-000487.supp1 Supplementary data

### Flow cytometry

At endpoints of 15 days following tumor induction, livers were excised as described earlier, and caudate lobes were separated for processing for flow cytometry. Tumors were macroscopically isolated from liver parenchyma, and both tumor and macroscopically tumor-free liver were treated with tissue digest medium (0.25 mg/mL Liberase, Sigma and DNAse, Sigma) at 37 degrees for 40 min. Tissues were then pushed through a stainless-steel mesh, treated with red blood cell lysis buffer, and resuspended in FACS wash buffer (10% bovine serum albumin/5 mM EDTA/0.01% sodium Azide in PBS PH 7). 1×10^6^ cells were stained with antibody cocktails directly conjugated to fluorochromes (CD45-BV510, CD3-PE, CD8-PECy7, PD-1-APC, CD4-APC-Cy7 (BD Bioscience) and CD103-FITC (Miltenyi biotec) and the viability dye, DAPI (additional file 1: online supplementary table 1). Each sample was analyzed on a FACS CANTO II (BD Biosciences) and data were analyzed by FlowJo™ v10. Lymphocytes were identified as live (DAPI negative), single cells that were negative for autofluorescence, CD45^+^ and CD3^+^ (online supplementary figure S2A). Fluorescence minus one (FMO) controls were used to accurately gate CD4^+^ or CD8^+^ T cells and then the expression of PD-1 and CD103 on these T cell subsets (online supplementary figure S2B), including CD3^+^ cells that were expressing neither CD4 nor CD8, termed double-negative T cells (DNTs).

### Statistical analysis

All data were presented as the mean value of each group ±SE of the mean. Correlation matrix was generated using correlation coefficient r (or rs) for each pair of variables: length (µm), area (µm^2^), TM positivity, tumor core positivity and total positivity. A heat map of R^2^ calculating p values (two-tail) was generated. Statistical analysis was conducted using GraphPad Prism V.8.3.0 (GraphPad Software, San Diego, California, USA) using both parametric and non-parametric analytical tests as appropriate. All statistical tests were two-sided, and a p-value of 0.05 was considered statistically significant.

## Results

### Captopril inhibits metastatic growth and reduces tumor viability

The stages of metastatic development of the CRLM model have been previously characterized (figure 1B).^28^ These experiments confirmed previous findings by this laboratory^18^ that captopril treatment has profound effects over metastatic growth after the metastases were fully established by 21 days following tumor induction (figure 1C, D). In this study, at day 15 endpoint, there was a trend in the reduction of tumor load by 3.94% difference. In contrast, liver collected at 21 days post-tumor induction from the group treated with captopril had significantly less tumor burden than the control (28.61% difference) (figure 1D). In this study, we also examined tumor viability as this is a better indication of treatment efficacy. Quantitation of live tumor area demonstrates that the percentage of viable tumor area in the captopril treatment group was significantly less, compared with the control group at 21 days post-tumor induction (figure 1E, F). Similar to the findings for tumor burden, while there is a trend, no significant differences in tumor viability could be determined at day 15 (figure 1F). Captopril treatment reduced the number of metastases, though this reduction did not reach any significance, the size of metastatic foci was in fact significantly reduced (online supplementary figure S3).

### Captopril treatment modulates spatial and temporal infiltration of tumor lymphocytes expressing CD3^+^ and CD4^+^

**Figure 2:**
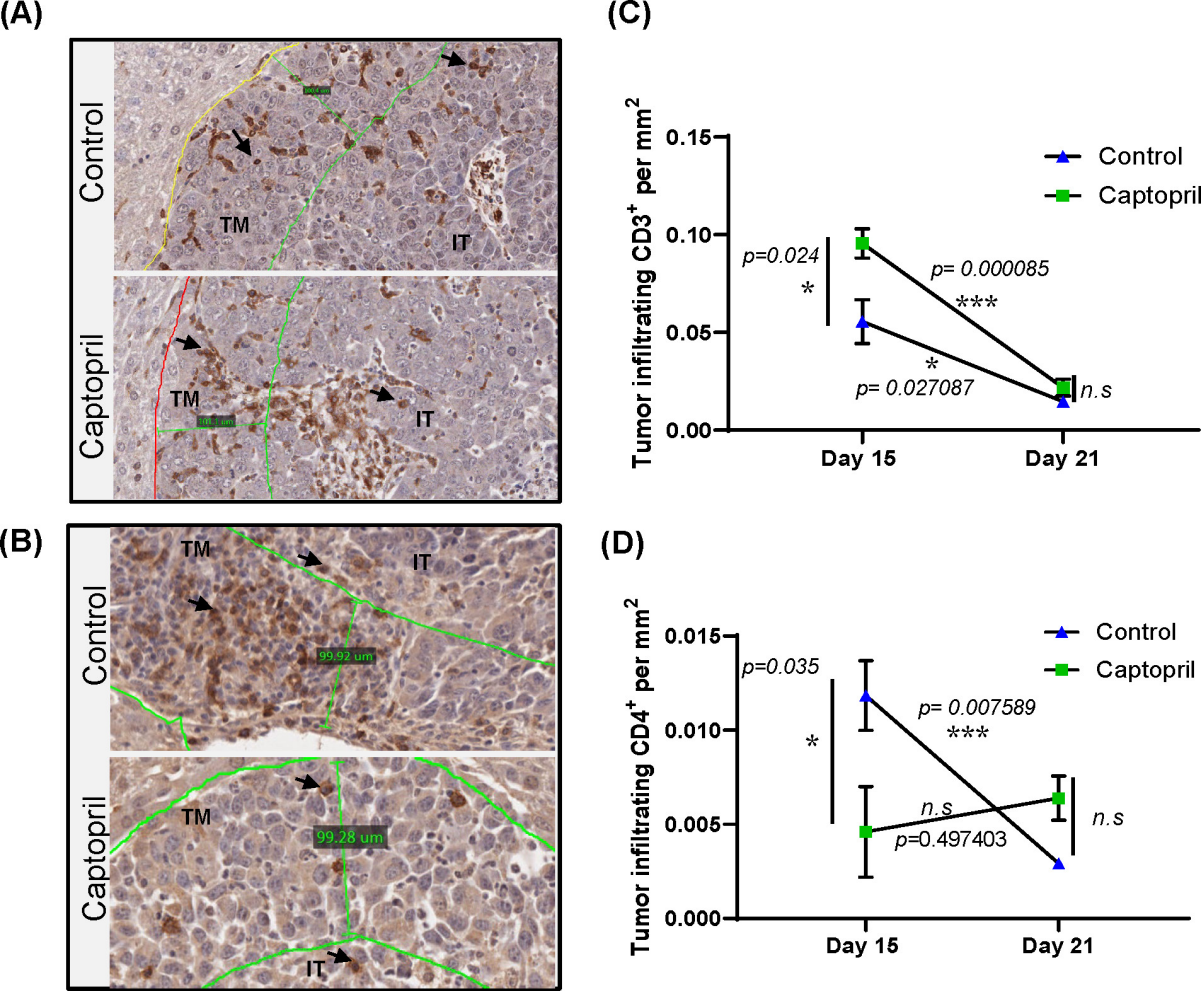
Tumor infiltrating lymphocytes are modulated by RASi. Representative microphotographs of histological quantitation of (A) CD3^+^ lymphocytes and (B) CD4^+^ lymphocytes in tumor tissues at day 15 (scale bar 100µm), including mark up for tumor margin (TM) and intratumoral (IT) areas. Quantitation of the total area staining positive for (C) CD3^+^ or (D) CD4^+^ as an area per mm^2^ of total tumor area, (excluding necrotic areas) for tissues collected at day 15 and day 21. Data n≥5 presented as mean ± SEM. Significantly different data represented by * p<0.05, ** p<0.01, and***p<0.001. n.s, not significant.

We used IHC to examine changes in CD3^+^ and CD4^+^ lymphocytes within the tumor tissues due to captopril treatment. Significant increases were seen in CD3^+^ lymphocytes in the treated group at day 15 (figure 2A, C). On the other hand, CD4^+^ lymphocytes were significantly decreased at this time point (day 15) as seen in figure 2B, D. Visual examination of the staining shows that lymphocyte infiltration tends to accumulate around the intratumoral vessels (figure 2A, B). Significant increases in the number of CD3^+^ lymphocytes were observed in both TM and intratumoral assessment in the captopril-treated group (figure 2A).

Interestingly, both density and distribution of CD3^+^ tumor-infiltrating lymphocytes inversely correlate with the area of liver metastases (online supplementary figure S4A). While the frequency of CD4^+^ cells in the control group significantly decreased in the same pattern as the CD3^+^ lymphocytes between the two time points; in the treated group, the CD4^+^ T cells did not significantly change compared with day 15, and in fact, at day 21, the frequency of CD4^+^ in the treated group is relatively higher than at day 15, but it is not significantly higher compared with that of the control at day 21 (figure 2C, D). These results suggest that the increased infiltration of CD3^+^ T cell population at day 15 was clearly not CD4^+^ T cells and that at day 21, these are no longer within the tumor in elevated numbers, giving impetus for a thorough investigation of day 15 T cell phenotype changes induced by captopril (figure 2).

### Captopril treatment does not alter the tumor PD-L1 expression

**Figure 3:**
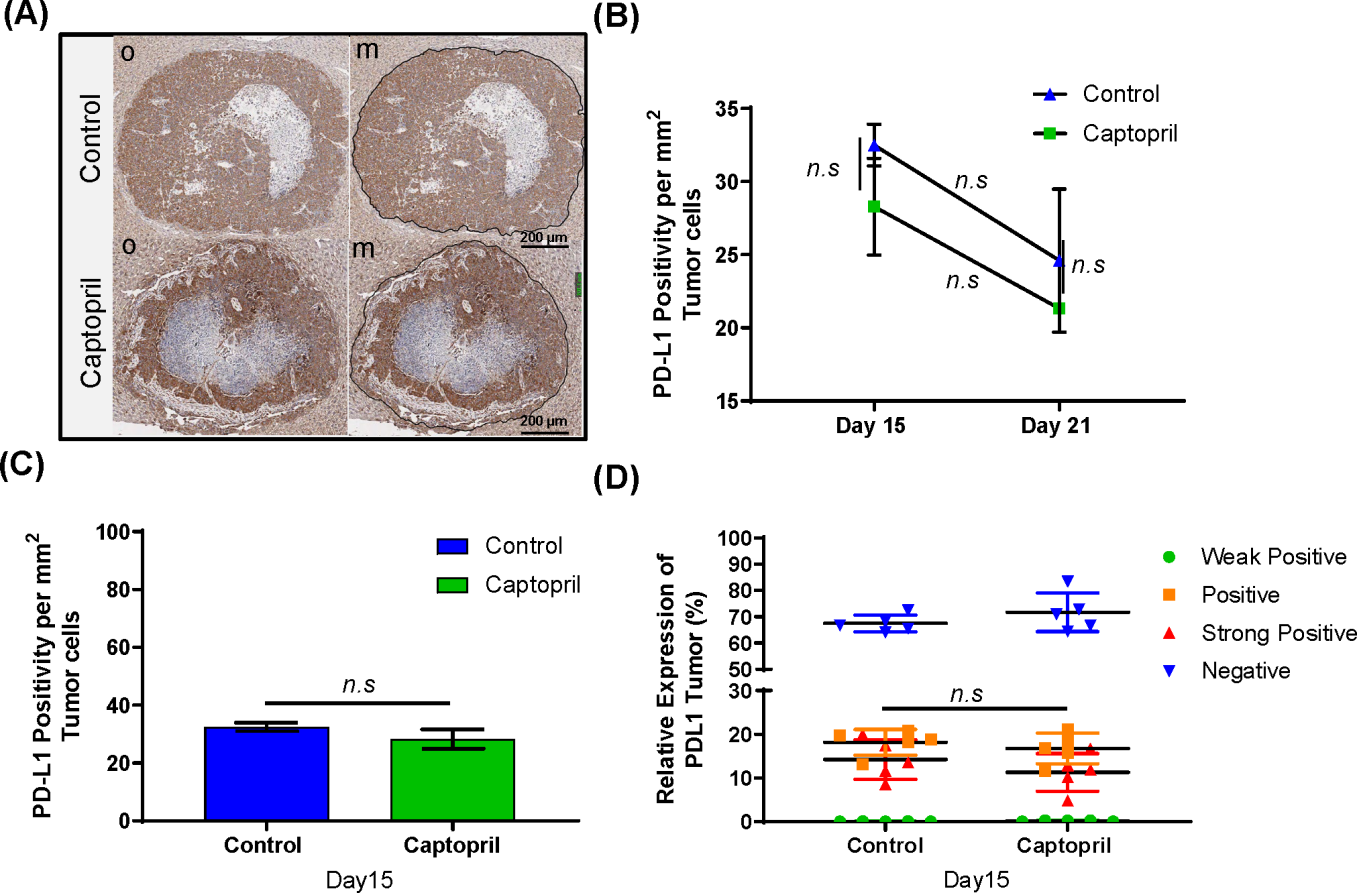
Temporal and spatial PD-L1^+^ expression in tumor cells. (A) Representative microphotographs of IHC PD-L1 staining; original (o) and marked-up (m) at day 15 post-tumor induction (black line) (Scale bar = 200 µm). (B) Histological quantitation of PD-L1 expressions at day 15 and 21 post-tumor induction. (C) Quantitation of the total tumor area staining positive for PD-L1^+^ as an area per mm^2^ of total tumor area, (excluding necrotic areas) for tissues collected at day 15. (D) PD-L1 expression in tumor cells comparison between four levels of staining are shown: weak positive (green), positive (orange), strong positive (red), and negative (blue). Datasets expressed as mean ± SEM with n ≥ 5 mice. n.s., not significant.

Using IHC we also investigated whether the tumor in our model expresses PD-L1 (figure 3A), and whether captopril treatment alters tumor PD-L1 expression. While there is a strong expression of PD-L1 in the tumor, this expression was not significantly altered at any time point due to the treatment (figure 3B) and was not tumor size dependent (figure 3C). No significant difference was found between different populations of tumor cells expressing PD-L1 at different intensities between the control group and captopril at day 15 (figure 3D).

### Captopril treatment differentially modulates T cell subpopulations infiltrating into the tumor and liver tissues

**Figure 4:**
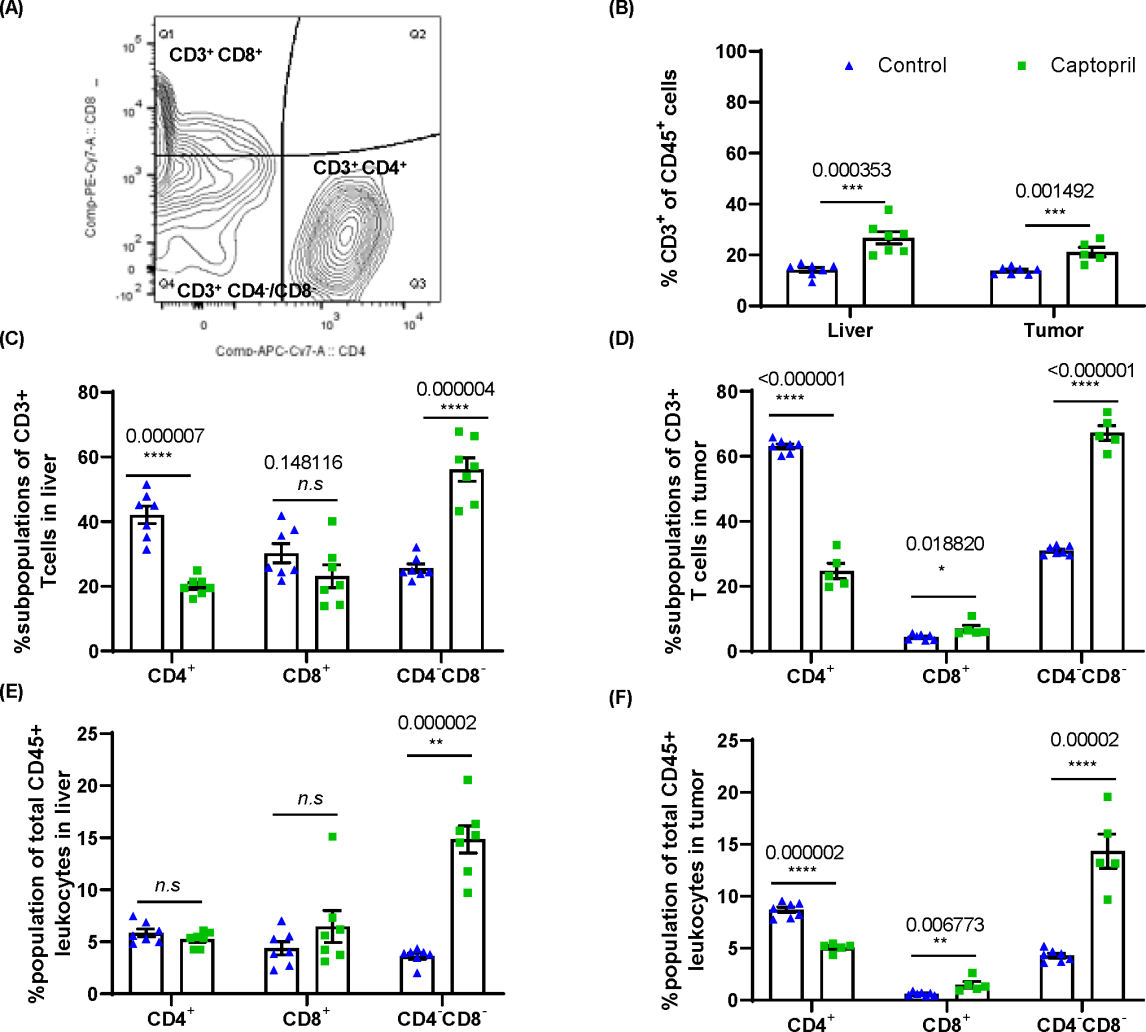
Renin–angiotensin system inhibitor differentially alters the proportions of CD3^+^ subpopulations of lymphocytes in the liver and tumor. (A) Gating strategy for analysis of CD3^+^CD4^+^, CD3^+^CD8^+^ and CD4^–^CD8^–^. (B) Quantitation of the proportion of CD3^+^ lymphocytes as a proportion of total CD45^+^ leukocytes in the liver and tumor. The proportion (%) of CD3^+^ lymphocytes expressing CD4, CD8 and those negative for both CD4 and CD8 (CD4^–^CD8^–^) in (C) the liver and (D) tumor. The proportion of each subpopulation within the total CD45^+^ leukocyte population in (E) the liver and (F) tumor. Data n≥5 are presented as mean±SEM; significantly different data are represented by *p<0.05, **p<0.01, ***p<0.001, and ****p<0.0001. n.s., not significant.

Flow cytometry analysis of dissected tumor tissues was used to identify the phenotype and the subpopulations of CD3^+^ T cells in tumor that were observed by IHC to be modulated by captopril at day 15 (figure 4A). We also analyzed the T cell subpopulation infiltration into liver tissues that had been macroscopically dissected from tumors. During liver harvesting, livers were perfused via portal vein cannulation with 20 mL of saline to remove peripherally circulating lymphocytes from the analysis; thus, data represent tissue-infiltrating cells.

FACS confirmed that captopril treatment significantly increased the infiltration of CD3^+^ T cells into the tumor. Interestingly, these changes were also seen within the liver parenchyma (figure 4B) indicating that CD3^+^ T cells, trafficking from circulation and/or dividing within the tissue in both liver and tumor, are affected by captopril.

The phenotype of CD3^+^ T cells was examined in terms of the proportion of these cells expressing CD4 and CD8 and those expressing neither CD4 nor CD8, termed DNT (figure 4C, D) and were found to be differentially modulated by captopril. Both in the tumor and liver tissues, there is a decrease in the proportion of CD4^+^ and an increase in the proportion of DNT. On the other hand, there is an increased proportion of CD8^+^ T cells in the tumor, while in the liver, this subpopulation decreases (figure 4C, D).

When considering these populations as a proportion of total leukocytes (CD45^+^) in these tissues, we find that in the liver, captopril treatment does not change either CD4^+^ or CD8^+^ subpopulations, whereas the DNT population is significantly increased (figure 4E). In the tumor, we also see a decrease in the CD4^+^ population while the CD8^+^ and DNT subpopulations significantly increased (figure 4F). This may indicate that in the liver tissue, the increase in CD3^+^ T cells induced by captopril is due to the infiltration of DNTs. Within the tumor, however, the changes induced by captopril are more complex and may reflect changes in the TME that affect the ability of each subpopulation to exist there.

### Captopril treatment differentially modulates the activation of T cell subtypes in the tumor and liver tissues

**Figure 5:**
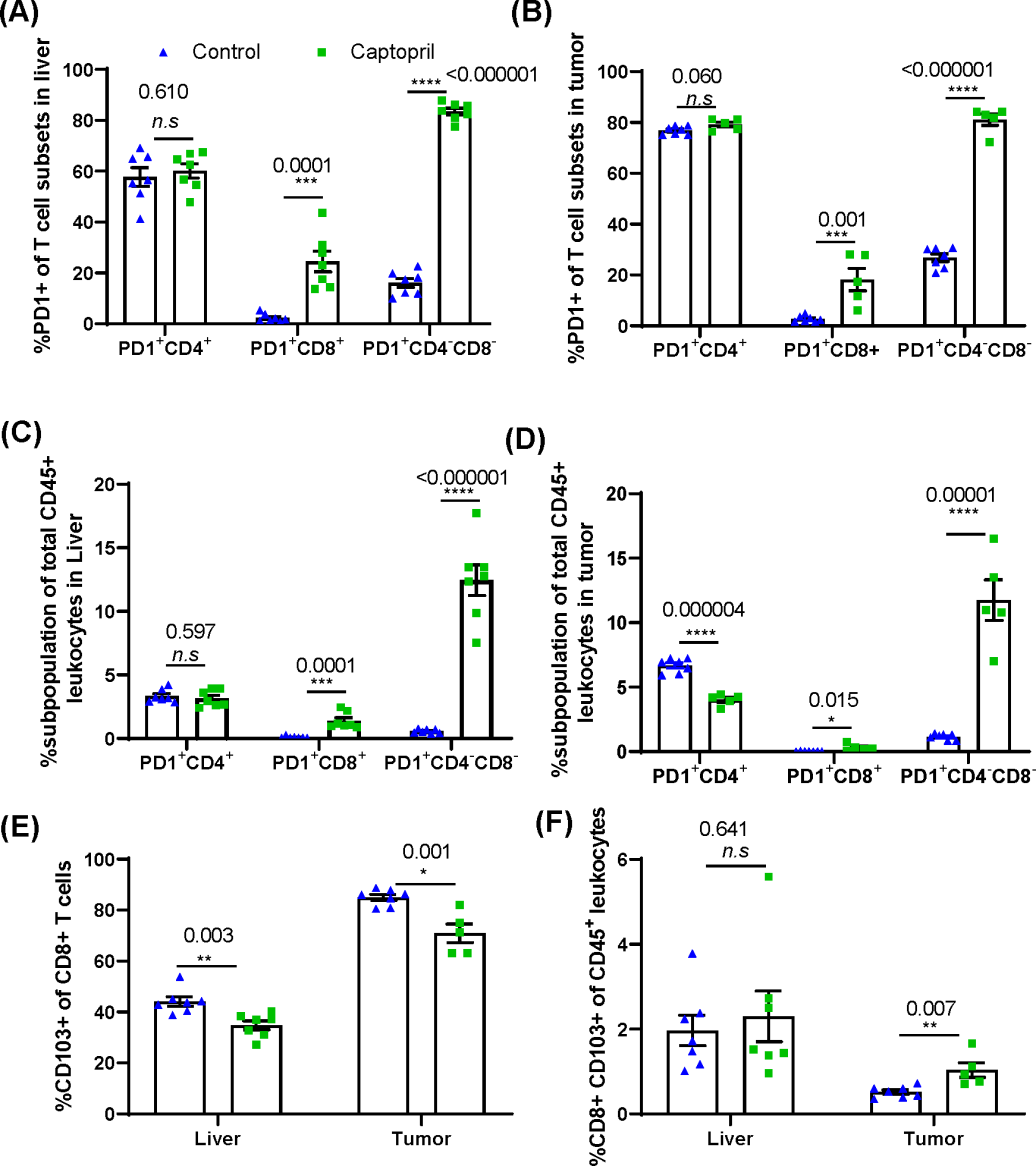
Renin–angiotensin system inhibitor differentially changes the proportions of CD3^+^ subpopulations expressing PD-1^+^ and tissue resident CD8^+^ lymphocytes in the liver and tumor. Quantitation of the proportion of each CD3^+^ subpopulation expressing PD-1^+^ in (A) the liver and (B) tumor. The percentage of PD-1^+^ lymphocyte subpopulations as a proportion of total CD45^+^ leukocytes in (C) the liver and (D) tumor. (E) Proportion of CD8^+^ lymphocytes expressing tissue resident marker CD103 in the liver and tumor. (F) The percentage of CD103^+^ CD8^+^ lymphocytes as a proportion total CD45^+^ leukocytes in the liver and tumor. Data n≥5 are presented as mean±SEM; significantly different data are represented by *p<0.05, **p<0.01, ***p<0.001, and ****p<0.0001. n.s., not significant.

To investigate changes in the activation state of the CD3^+^ T cell subtypes, the cells were also stained for expression of Programmed cell death-1 (PD-1), an inhibitory receptor upregulated following T-cell activation by cognate antigen and is specific for checkpoint ligand PD-L1. Captopril treatment increased the proportion of CD8^+^ and DNT subpopulations expressing PD-1 in both liver and tumor (figure 5A, B).

When considering these activated populations as a proportion of total leukocytes (CD45^+^) in these tissues, we find that in the liver and tumor tissues, captopril treatment again increases activated CD8^+^ and DNTs; however, in the tumor, the proportion of total leukocytes that is activated CD4^+^ significantly decreases (figure 5C, D). This may indicate a role for captopril in the activation of CD8^+^ and DNT but also in suppressing the activation of CD4^+^ T cells within the tumor.

We further investigated the proportion of CD8^+^ T cells expressing CD103 to delineate the effect of captopril on the tissue resident CD8^+^ T cell (T_RM_) population. The percentage of CD8^+^ T cells expressing CD103^+^ was significantly reduced in both liver and tumor tissues in the treated group (figure 5E). However, within the total leukocyte population, the proportion of CD8^+^ CD103^+^ T cells was significantly increased in tumor and was not affected in the liver (figure 5F).

This suggests a potential mechanism of how captopril may control tumor growth by improving the cytotoxic T cell response.

## Discussion

Numerous retrospective clinical studies acknowledge significant improvement in overall survival, progression-free survival, and disease-free survival in patients with cancer using RASi as antihypertensive medication.^30^ There is a clear upregulation of the RAS component, AT1R within the adenoma–colorectal–liver metastasis axis.^31 32^ Experimental and clinical studies over the last decade indicate that RASi impairs tumor progression through modulation of the tumor vasculature^33 34^ and tumor desmoplasia^35^; thus far, its role in tumor immunoregulation is not yet clear. Almost all immune cells express components of the RAS pathway, and several studies implicate a role for RAS in immune dysregulation in several chronic and autoimmune diseases.^36^ Our previous findings show that the RASi, captopril, reduced tumor inflammation^18^ and temporally modified the frequency of TAMs within the TME.^25^ Additionally, macrophage depletion experiments show that TAMs are important for RASi control of tumor.^25^

In this study, we investigated the temporal dynamic changes of specific T lymphocyte subpopulations, in response to captopril treatment and their trafficking patterns into the liver and tumor tissues. Captopril treatment significantly and temporally increased CD3^+^ T lymphocyte infiltration both into the tumor and the liver. This finding suggests that increased infiltration of T cells into the tumor may have a role in the observed tumor inhibition as clinical studies report a positive correlation between tumor lymphocyte density and survival outcome.^37^ The greatest observed changes were seen at day 15 post-tumor induction, possibly reflecting a timely development of antitumor responses and the effect of captopril treatment. By day 21, the infiltration decreased and the significance of this is not clear as the subpopulation composition was not analyzed at this time point. RASi reduces the immunosuppressive TME by altering the activity of TAMs, MDSCs and CAFs,^27^ and this may account, at least in part, for the increase in CD3^+^ T lymphocyte infiltration observed in this study.

In these experiments, the effect of RASi on the infiltration of T cell subtypes appears specific and possibly local to tumor-affected tissues. It is important to note that the T cells infiltrating the liver may provide a substantial pool, along with those from the peripheral circulation that would be infiltrating the tumor. Remarkably, we found the inhibition of RAS markedly attenuated tumor-infiltrating CD4^+^ T cells. AT1R stimulation has been shown to play a role in the differentiation of CD4^+^ T cells into Th1 cell through the modulation of proteasome function; thus, RASi may inhibit this mechanism of CD4^+^ T cell antigen-mediated activation.^38^ The proportion of activated circulating CD4^+^ T cells may have been diminished as a result of reduced AngII; therefore, trafficking into the liver and tumor was profoundly affected, as it was reported during deoxycorticosterone acetate–salt hypertension.^39^

RASi appears to not have a selective effect on the infiltration of CD8^+^ T cells into the liver; however, the significant increase in CD8^+^ lymphocytes into the tumor, particularly those that are identified as tissue resident (CD103^+^) and PD-1^+^, may indicate a recently activated tumor-specific cytotoxic T cell population. Studies reported a positive correlation of CD8^+^-infiltrating lymphocytes and control of tumor growth.^40^ Additionally, the intratumoral presence of T cells expressing the integrin CD103 has been strongly correlated with favorable prognosis for patients with cancer,^41^ independently of the infiltration of CD8^+^ T cells.^42^ Moreover, T_RM_ cell express immune checkpoint molecules, which may be a potential surrogate marker of response to immunotherapy as they could expand early during anti-PD-1 treatment.^43^

Unexpectedly, the most responsive subpopulation of immune cells to captopril treatment was the DNTs. CD3^+^ lymphocytes that do not express CD4 and CD8 may include TcR alpha/beta (TcRαβ^+^) and TCR gamma/delta (TcRγδ^+^); however, at this stage, identification of the type of DNT has not been examined in this study. DNTs represent only a small fraction of the total peripheral lymphocyte composition (1%–3%) in healthy subjects.^44^Nevertheless, they are reported to increase in certain diseases with context-specific immune roles such as immune inflammatory and disease-exacerbating activities in autoimmune diseases^45^ to anti-inflammatory^46^ or proinflammatory roles in tissue injury and ischemia.^47^

Interestingly, DNTs have been reported to be present in several solid cancers (melanoma, renal cell carcinoma, and glioblastoma) and to increase on treatment.^48^ The phenotypes of these cells are cancer-specific and distinct from that of resting DNT, but closely resembling DNT in other cancer types and across species.^48^ Furthermore, they positively correlate with treatment outcome, in concordance with the findings in this study.^48^ DNTs have been previously reported to contribute to antimelanoma immunity.^49^ In addition, this immunomodulatory effect is congruent with the reduced frequency of CD4^+^ T cell subpopulation as DNTs have been shown to suppress metabolic activation of CD4^+^ T cells (53) and could account for the CD4^+^ T cell reduction in this study. Nevertheless, the contribution of DNTs to antitumor immunity has only begun to be considered.

In the meantime, cancer immunotherapy strategies have mainly focused on modulating the function of infiltrating CD4^+^ and CD8^+^ lymphocytes. The presence and quality of the immune cell infiltrate of the tumor have been shown to be of prognostic potential for the response of different tumors to immunotherapies, regardless of tumor stage.^37^ However, when PD-L1 blockers are used, a selective expansion of tumor-infiltrating CD4^+^ and CD8^+^ T cells expressing both activating (inducible T cell costimulator (ICOS, CD278)) and inhibitory (Lymphocyte activation gene 3 (LAG-3) and PD-1) molecules is seen.^40^

This study showed significant upregulation in PD-1^+^ expression on both the CD8^+^ and DNT tumor-infiltrating cells in the captopril-treated mice. A recent study reported that DNT infusion, in combination with anti-PD-1, resulted in increased DNT-mediated antitumor activity during in vivo treatment of lung cancer.^49^ Nakamura *et al*, in the only other *in vivo* study on the RASi immunoregulatory effects, report that RASi, in combination with PD-L1 inhibitors, improves the effect on tumor inhibition.^27^ The increase of lymphocyte populations expressing PD-1 within the RASi-treated tumors, as seen in our study, opens up an opportunity to combine RASi with a combination of checkpoint inhibitor treatments that may able to increase the efficacy and durability of the antitumor response.^40^

Humans and outbred animal species display wide variability in the antitumor immune responses and drug treatments. In addition, to the genetic contribution, numerous studies report a strong sexual dimorphism in the immune responses.^50^ Furthermore, studies also indicate that some components of the RAS pathway display a sex-biased differential expression in certain tissues that may lead to gender biased response to RASi treatments as well.^51^ This study used inbred single-strain male mice to overcome genetic and sexual dimorphism variabilities. However, immune responses to RASi treatments for CRLM need to be investigated in additional animal models of both genders and in clinical studies. Elucidation of genetic and gender differences to RASi response will further define the appropriate patient cohort, thus tailoring treatment to those likely to benefit.

## Conclusion

This study described the immunomodulatory effect of RASi using captopril, comparing an early and a later stage of tumor development. Treatment significantly increased the infiltration of CD3^+^ T cells into the liver and tumor and altered the phenotypical composition of infiltrating CD4^+^ and CD8^+^ T cells, and DNTs. Additionally, captopril treatment increased the expression of checkpoint receptor PD-1 on the CD8^+^ and DNTs. The significance of these changes needs to be further characterized, especially the identity and function of the DNT population.
