# Supplementary material for: Immunomodulatory effects of renin–angiotensin system inhibitors on T lymphocytes in mice with colorectal liver metastases
Source: J Immunother Cancer. 2020 May 24;8(1):e000487. doi: 10.1136/jitc-2019-000487 (PMC7253054; doi:10.1136/jitc-2019-000487)
Supplement: Supplementary data [file jitc-2019-000487supp001.pdf]

SUPPLEMENTARY FIGURES- Dora et. al (2020)

Table S1. FACS panel for mouse studies.

CD marker	fluorophore	clone	Brand	cat #
CD3e	PE	145-2c11	BD pharmingen™	553064
CD4	APC cy7	GK1.5	BD pharmingen™	561830
CD8a	PE cy7	53-67	BD Biosciences	561097
CD103	FITC	2 E 7	Miltenyi Biotec	130-118-681
CD279 (PD1)	APC	REA802	Miltenyi Biotec	130-111-801
CD45	BV510	30-F11	BD Horizon	563891
CD16/32	pure	clone93	Miltenyi Biotech	130-092-574

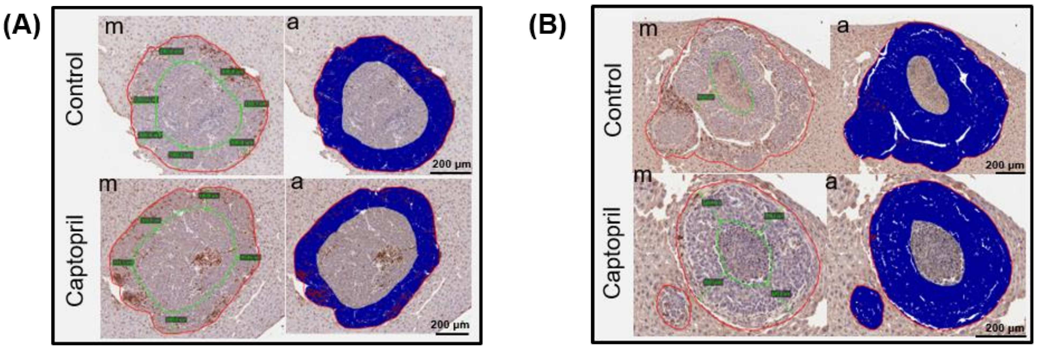

Fig. S1 Histological quantitation strategy. **(a)** representative microphotographs of IHC CD3 staining; **(b)** representative microphotographs of IHC CD4 staining; marked-up (m) and annotated (a) tumor margin (red line) and intratumoral (green line) distribution at day 15 post-tumor induction (Scale bar = 200 μm).

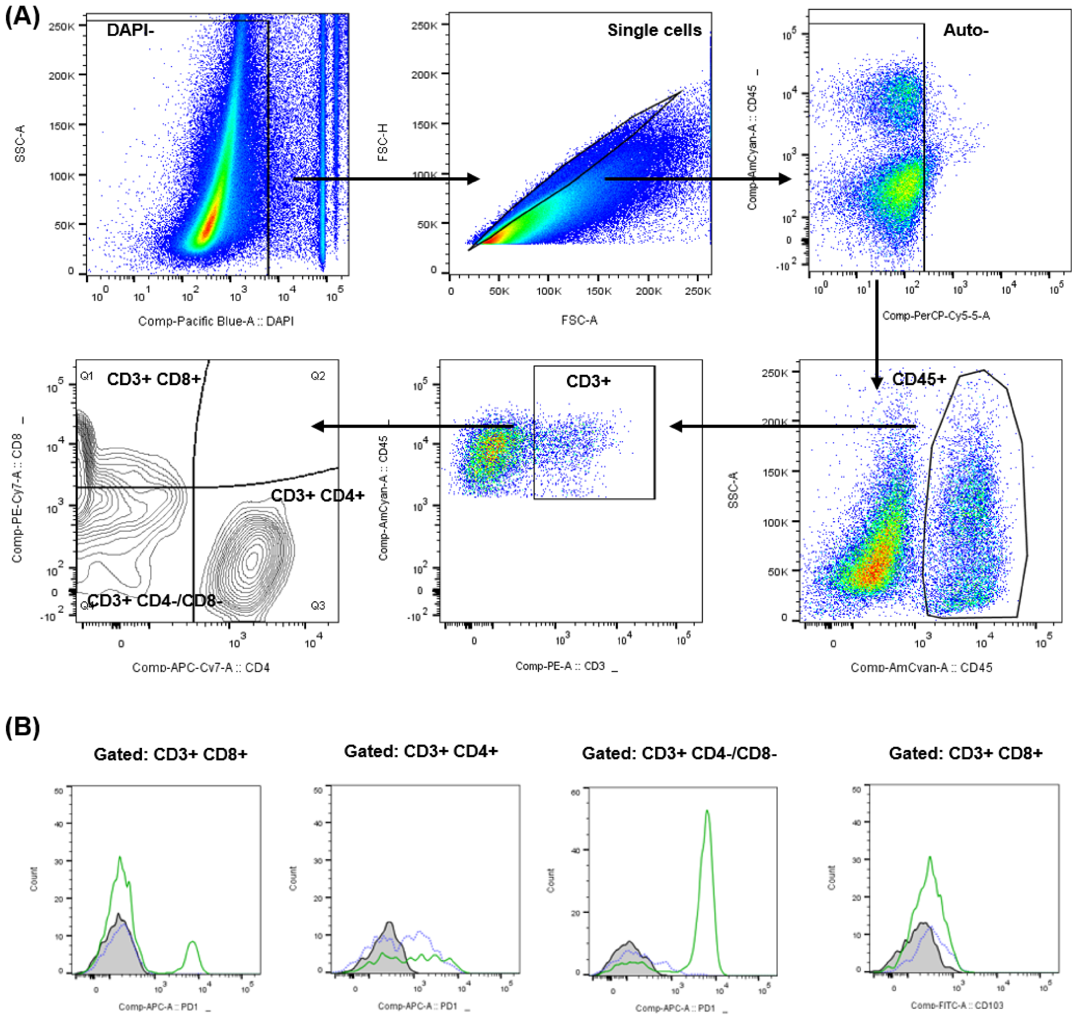

Fig. S2 Gating strategy for analysis of lymphocyte subpopulations. **(a)** Viability, single cells and auto fluorescence were used as optimising strategies to analyse the CD3⁺ lymphocyte subpopulations within the total CD45⁺ leukocyte population in liver (example given) and tumor. **(b)** The expression of PD1 and CD103 on gated lymphocyte subpopulations on tissues from captopril treated (green line), saline treated (blue dash) and unstained for PD1 and CD103 controls (grey fill).

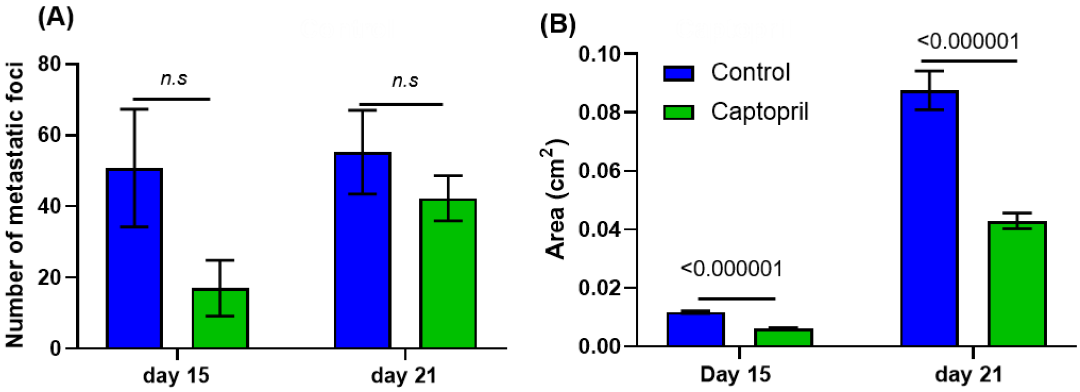

Fig. S3 Captopril treatment effect the number and size of metastatic foci. **(a)** Number of metastatic foci in the control and captopril treatment group at day 15 and day 21. **(b)** Tumor region area (cm) of control and captopril treated group at day 15 and day 21. Datasets expressed as mean ± SEM with n ≥ 5 mice for each group.

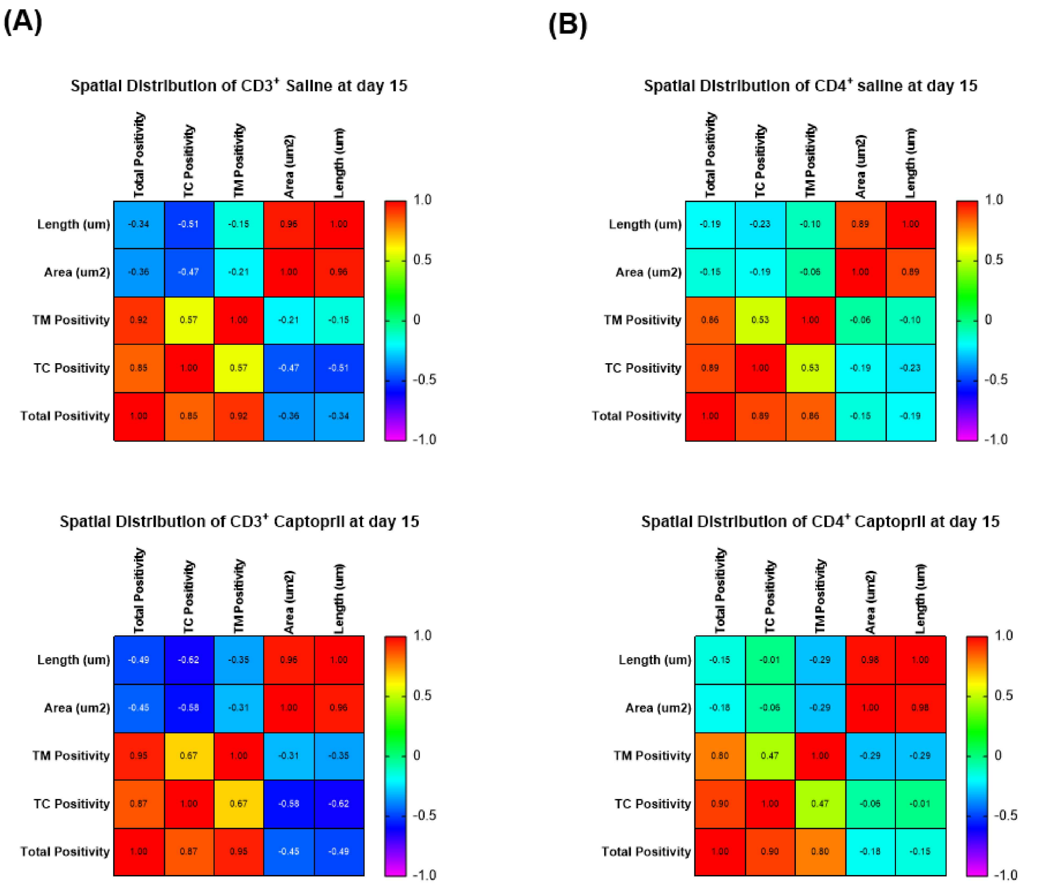

Fig. S4 Correlation matrix using Pearson r to determine the distribution of tumor infiltrating **(a)** CD3$^+$ and, **(b)** CD4 $^+$ lymphocytes within inner tumor invasive margin and intratumorally. coefficient r (or rs) are shown for each pair of variables: length (µm), area (µm^2), tumor margin (TM) positivity, tumor core (TC) positivity and total positivity. A heat map of R^2 calculating P values (two-tail) was generated. Datasets expressed as mean ± SEM with n ≥ 5 mice for each group (equivalent >100 tumor regions in each group) excluding tumor region values outliers ROUT (Q=1%). Significantly different data is represented by * ($p<0.05$).
